# Prediction of Propulsion Kinematics and Performance in Wheelchair Rugby

**DOI:** 10.3389/fspor.2022.856934

**Published:** 2022-07-07

**Authors:** David S. Haydon, Ross A. Pinder, Paul N. Grimshaw, William S. P. Robertson, Connor J. M. Holdback

**Affiliations:** ^1^South Australian Sports Institute, Kidman Park, SA, Australia; ^2^Faculty of Sciences, Engineering, and Technology, University of Adelaide, Adelaide, SA, Australia; ^3^Paralympic Innovation, Paralympics Australia, Adelaide, SA, Australia; ^4^College of Health and Life Sciences, Hamad Bin Khalifa University, Doha, Qatar

**Keywords:** paralympic sport, wheelchair propulsion, wheelchair configuration, regression, modeling

## Abstract

Prediction of propulsion kinematics and performance in wheelchair sports has the potential to improve capabilities of individual wheelchair prescription while minimizing testing requirements. While propulsion predictions have been developed for daily propulsion, these have not been extended for maximal effort in wheelchair sports. A two step-approach to predicting the effects of changing set-up in wheelchair rugby was developed, consisting of: (One) predicting propulsion kinematics during a 5 m sprint by adapting an existing linkage model; and (Two) applying partial least-squares regression to wheelchair set-up, propulsion kinematics, and performance. Eight elite wheelchair rugby players completed 5 m sprints in nine wheelchair set-ups while varying seat height, seat depth, seat angle, and tire pressure. Propulsion kinematics (contact and release angles) and performance (sprint time) were measured during each sprint and used for training and assessment for both models. Results were assessed through comparison of predicted and experimental propulsion kinematics (degree differences) for Step One and performance times (seconds differences) for Step Two. Kinematic measures, in particular contact angles, were identified with mean prediction errors less than 5 degrees for 43 of 48 predictions. Performance predictions were found to reflect on-court trends for some players, while others showed weaker prediction accuracy. More detailed modeling approaches that can account for individual athlete activity limitations would likely result in improved accuracy in propulsion and performance predictions across a range of wheelchair sports. Although this would come at an increased cost, developments would provide opportunities for more suitable set-ups earlier in an athlete's career, increasing performance and reducing injury risk.

## Introduction

Current procedures for prescribing wheelchair set-up parameters such as seat height and seat angle are limited in wheelchair sport, relying on previous coach and player experience (Mason et al., 2013), optimizing parameters in isolation (Vanlandewijck et al., 2011; Mason et al., 2012, 2015), or requiring substantial amounts of testing (Usma-Alvarez et al., 2014; Haydon et al., 2019). These issues stem from difficulties in: monitoring on-court performance, where inertial measurement units (IMUs) only recently provide a reliable solution (Pansiot et al., 2011; van der Slikke et al., 2015, 2016; Shepherd et al., 2016); the substantial cost associated with wheelchair purchase (often $5,000–$10,000 USD); adjusting wheelchair set-ups on current wheelchairs; and optimization that varies for individual players, where a greater focus on individual impairments could potentially improve the ability to quickly achieve near optimal set-ups.

In wheelchair rugby (WCR), players are assigned point classification scores ranging from 0.5 to 3.5 points depending on their sport specific activity limitation (the ability to perform key tasks within the sport with regards to their impairment) where a lower score indicates greater limitation (International Paralympic Committee, 2020). The classification process considers trunk, arm, and hand function [where “function” includes strength, range of motion and co-ordination (Haydon et al., 2018a)] and hence players with varying impairment types [i.e., impaired muscle power—potentially due to spinal cord injuries (SCI)—or limb deficiencies which can be congenital or due to amputation] can be assigned the same classification scores. Optimizing wheelchair set-up based on either classification or impairment type is therefore not viable (Haydon et al., 2019; International Wheelchair Rugby Federation, 2021). Hence methods are needed that can provide detailed quantitative (and individualized) insights into the effects of set-up parameters on performance factors, while also minimizing the amount of time and effort of on-court testing.

Ideally, on-court testing would be used for optimizing wheelchair configurations, where athletes can be tested under conditions that are representative of competition demands as far as practically possible (Goosey-Tolfrey and Leicht, 2013). This testing can reveal significant differences in performance for set-up parameters such as wheel camber angle (Mason et al., 2012), seat angle and depth (Haydon et al., 2019), and even glove type (Mason et al., 2009). Small changes to some parameters can have substantial impacts on performance and on-court results, with the difference between executing or missing blocks on opposition dependent on just centimeters of position (Haydon et al., 2018a). However, despite the use of improved sensor technology and algorithms (combined with high-speed video) to identify key features of performance (Haydon et al., 2018a), on-court assessments remain difficult. This is due to the number of possible set-up parameters and combinations, with each of these having various effects on acceleration, agility, and ball-handling (Mason et al., 2010, 2013). Achieving a balance across the range of set-up parameters (seat height, seat angle, etc.) and performance measures (acceleration, agility, etc.) becomes even more difficult when considering the trade-off for various parameters on performance, as well as the interaction between various parameters (Mason et al., 2010, 2013). To address this problem, a substantial time commitment is required from athletes and coaches for both testing and results interpretation (van der Slikke et al., 2016; Haydon et al., 2019) which also has limitations due to skill adaptation and preferences of athletes based on their previous experiences (Haydon et al., 2018b, 2019). Further developments are therefore desired in maximizing efficiency in optimizing wheelchair set-ups at an individual level; propulsion modeling provides a potential method to achieve this.

Most current wheelchair propulsion modeling approaches have focused on musculoskeletal models attempting to quantify shoulder loads in daily propulsion to assess or reduce the likelihood of shoulder injuries (Morrow et al., 2010; Rankin et al., 2012; Slowik and Neptune, 2013; Hybois et al., 2018; Lewis et al., 2018). This is clearly a crucial area for improving the well-being of wheelchair users, but it is unable to address performance aspects such as sprint or agility times. Due to the complexity of musculoskeletal models, creating valid individual representations of anthropometrics and muscular function is also an extensive process (Dembia et al., 2020; McErlain-Naylor et al., 2021). To address this, a linkage model has previously been developed that is able to predict changes in propulsion kinematics (contact and release positions) for changing seat height [the vertical distance from the rear of the seat to main (rear) wheel axle] and seat depth (often referred to as fore-aft position, the horizontal distance from rear of the seat to main (rear) wheel axle) during daily propulsion (Richter, 2001; Leary et al., 2012). It should be noted that these terms are used as they were clearly understood by the coach and participants involved, as well as aligning with previous literature, but in some cases do not conform to ISO standards (Waugh and Crane, 2013)—care should be taken to interpret these measures correctly. This method appears to be a more realistic solution for optimizing wheelchair set-up for performance due to the reduced time requirements and ease of adjusting for individual players. However, assessing the relationship between kinematics and on-court performance measure is difficult, particularly when this relationship with performance varies across players (Fletcher et al., 2021).

The development of regression approaches, such as partial least squares (PLS), provide a potential method for quantifying the relationship between wheelchair set-up, propulsion kinematics, and performance. These regression approaches consider several predictor variables (such as wheelchair set-up, or propulsion kinematics) and construct new predictor variables or components. These predictor components can then be used to estimate performance factors such as sprint time. Regression approaches attempt to find a relationship between the predictor variables and the predicted variable by minimizing the error across all conditions (Schumann et al., 2013). The PLS approach does this by linking the variability of predictors with the response through a simultaneous decomposition of all variables (Schumann et al., 2013). Such approaches have been used across a range of areas, including the design of running shoes and emotional reaction of consumers (Shieh and Yeh, 2013), pelvic shape prediction (Schumann et al., 2013), determination of sport rock climbing performance (Mermier et al., 2000), and technique analysis in sports (Federolf et al., 2014; Gløersen et al., 2018).

The aim of the current study was to investigate the ability of a PLS approach to predict sprint performance based on individual wheelchair set-up and predicted propulsion approaches. A subsequent aim was to assess the prediction accuracies of propulsion kinematics of a linkage model in comparison to measured propulsion kinematics. To achieve these aims, a linkage model was implemented to predict alterations in propulsion kinematics with changing wheelchair set-up for elite WCR players, and then use a PLS approach to predict the effect of these alterations on sprint performance. This work is intended as an exploration to determine if there is scope to expand research in this area rather than a validation of this approach. Using this simplified model (in comparison with musculoskeletal modeling), it was expected that prediction of propulsion kinematics with changing wheelchair set-up would be successful, and subsequently be able to infer performance measures (sprint time). The ability to predict performance measures is expected to link closely with the ability to predict propulsion kinematics.

## Method

### Participants

Eight elite WCR players were recruited and provided informed, written consent before completing testing. All players were members of the Australian WCR team, were classified by the International Wheelchair Rugby Federation (IWRF) and completed testing in an adjustable wheelchair using 25-inch wheels. Individual player details are summarized in Table 1.

**Table 1:** Player information, including impairment, classification, and experience information. Contact Prediction Method refers to whether these players required an alteration to the equations for calculating their kinematics (see Section Propulsion Prediction).

**Player**	**Impairment**	**Classification score**	**International experience (years)**	**Contact prediction method**
1	Limb deficiency	3.5	14	Altered
2	Limb deficiency	3.5	6	Original
3	Limb deficiency	3.5	3	Altered
4	Impaired muscle power	2.0	3	Original
5	Limb deficiency	2.0	1	Altered
6	Impaired muscle power	2.0	10	Original
7	Impaired muscle power	2.0	12	Original
8	Impaired muscle power	1.0	8	Original

### Testing

**Figure 1:**
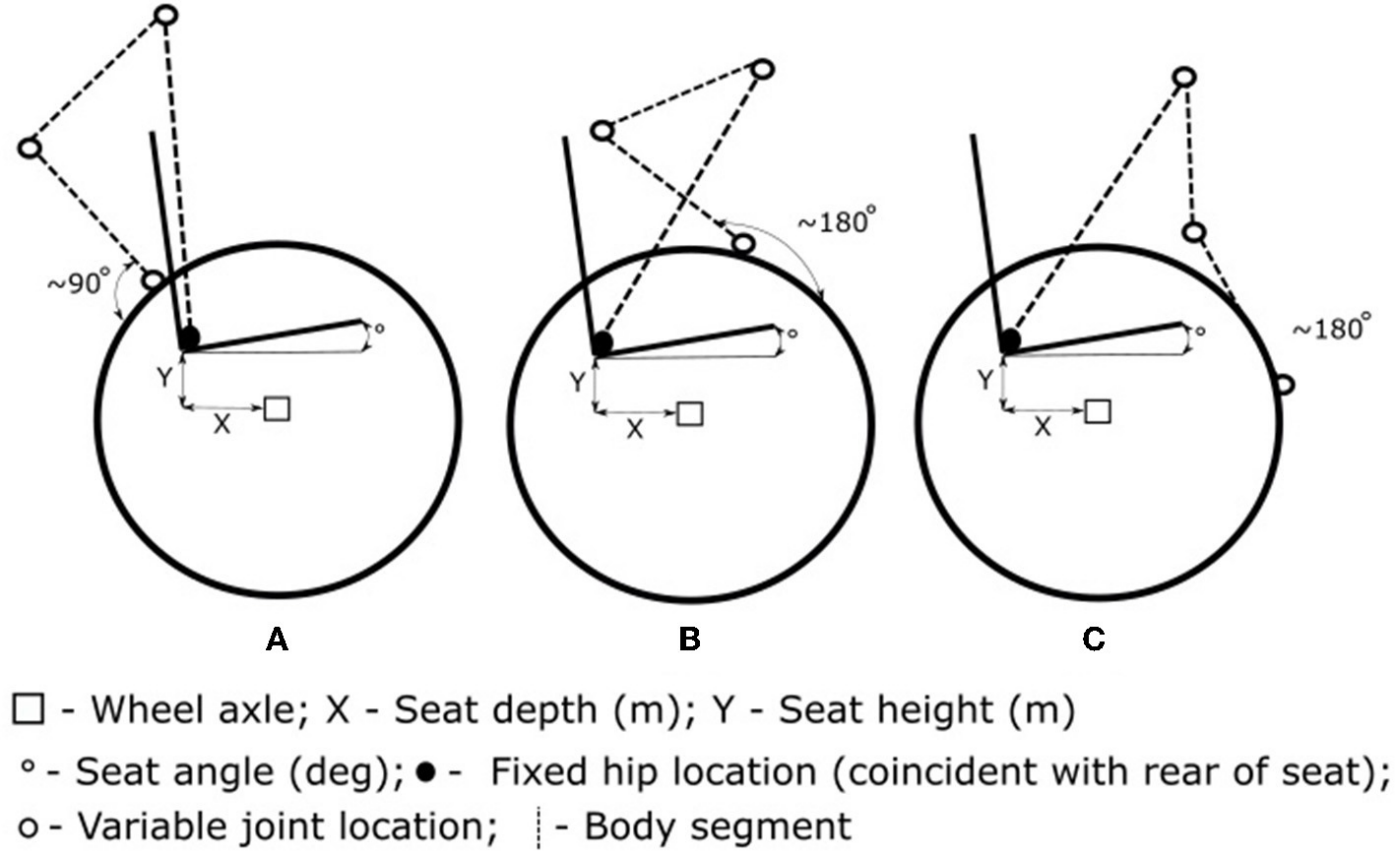
The propulsion model consisted of a trunk, upper arm, and forearm segments with a fixed hip position and variable seat height [the vertical distance from the rear of the seat to main (rear) wheel axle], seat depth [often referred to as fore-aft position, the horizontal distance from rear of the seat to main (rear) wheel axle], and seat angle (angle of the seat above the horizontal). Contact angle estimation varied between the previous assumption of the forearm being perpendicular to the wheel tangent at contact **(A)**, and an altered propulsion method where the forearm is close to parallel with the wheel tangent **(B)** at contact (Leary et al., 2012) for the added assumption. Release angle **(C)** is also presented for comparison with the contact positions, with assumption that release occurs when the forearm is parallel to the wheel tangent when the trunk is in its most flexed position. The propulsion kinematic angles (contact and release) are measured with respect to the location about top dead center of the wheel. The hip position visually presented here does not intend to represent the actual hip position for athletes in wheelchair rugby, with the model assuming that hip location is coincident with the rear corner of the seat^*^.

Testing consisted of an orthogonal design approach using an adjustable wheelchair. Orthogonal design is a robust design approach that reduces the time and cost associated with optimizing parameters in real-world applications (Mori and Tsai, 2011). Using an orthogonal array reduces the number of tests required by systematically varying the combinations of parameters and levels while maintaining the ability to identify the effects of specific parameter levels. After testing has been completed, level averages from each parameter (e.g., reduced seat height) are compared against the grand average to determine the effect of each parameter level (Mori and Tsai, 2011). This approach allowed for the variation of four set-up parameters (seat height, seat depth, seat angle, and tire pressure) at three levels (player's current level, an increase, and a decrease) using an L9 orthogonal array (9 total set-ups). Seat height and seat depth used the definitions described above, while seat angle as the sagittal angle of the seat above the horizontal—these are shown in Figure 1. Seat height and seat depth were adjusted by ±15 mm, seat angle by ±5 degrees, and tire pressure by ±15 psi. An example of the orthogonal design is provided in Supplementary Material. Players completed a warm-up and familiarization process in each set-up before completing two sprints while monitoring performance measures and propulsion kinematics. The 5 m sprint which was conducted from standstill in the athlete's own time with sprint time recorded using laser timing gates (SpeedLight, Swift Performance). All testing (including the athlete's current set-up) was performed in an adjustable wheelchair (mass of 14 kg), with the athlete using their own wheels and gloves, and strapping was consistent across trials. The set-ups were tested in a randomized order, including a set-up that replicated the players typical set-up. For more details on testing implementation and analysis, see Haydon et al. (2016).

Propulsion kinematics (contact and release angles) and performance time for the 5 m sprints, along with the set-up information, were monitored for the first three strokes due to their importance on WCR performance (West et al., 2014). Angles projected in the sagittal plane were calculated from digital footage (120 Hz, Go Pro Hero 3+, California, U.S.) that was analyzed as part of a custom Matlab (Mathworks, 2017b) script. The points of contact and release were identified by acceleration spikes from inertial measurement units (IMUs) located on each wheel (recording at 500 Hz, IMeasureU, NZ). The IMUs were secured to the outside of the disc wheel using tape in a location that avoided any interference during the stroke, with this resulting in the distance from the axle varying for each player. When these acceleration spikes were selected, the corresponding GoPro video frame (and ±2 frames either side) were prompted, with the user then visually confirming the contact or release moment. The propulsion kinematics were then measured in the sagittal plane view by selecting: (i) the center of the wheel as a reference point, (ii) a point directly superior to this in the digital video frame to attain the vertical direction, and (iii) the position of the hand on the pushrim/wheel. Hand landmarks differed between athletes due to limb impairments and variations in propulsion technique. Additionally, as seat angle has previously been linked to trunk motion (Vanlandewijck et al., 2011), trunk angles at contact and release for each of the first three strokes were investigated for the various seat angle levels. Trunk angle was determined using a similar method by selecting: (a) an approximate hip position (identification of hip position varied across players due to wheelchair design resulting in occlusion; landmarks specific to each player were used) as a local reference point, (b) a point directly superior to this in the digital video frame, and (c) the acromion to determine the trunk angle. Refer to Figure 1 for model representation of the hip and acromion positions. Note, the flexed trunk position was defined as a positive trunk angle. These results were then used as the input for each player's trunk angle in the linkage model, depending on the seat angle level. The intra- and inter-evaluator reliability of kinematic analysis was assessed across 20 trials by the lead researcher 2 weeks after initial analysis, and by an additional researcher, with good-to-moderate results of 2.6–9.7% technical error of measurement across all variables (Duthie et al., 2003).

### Modeling

**Figure 2:**
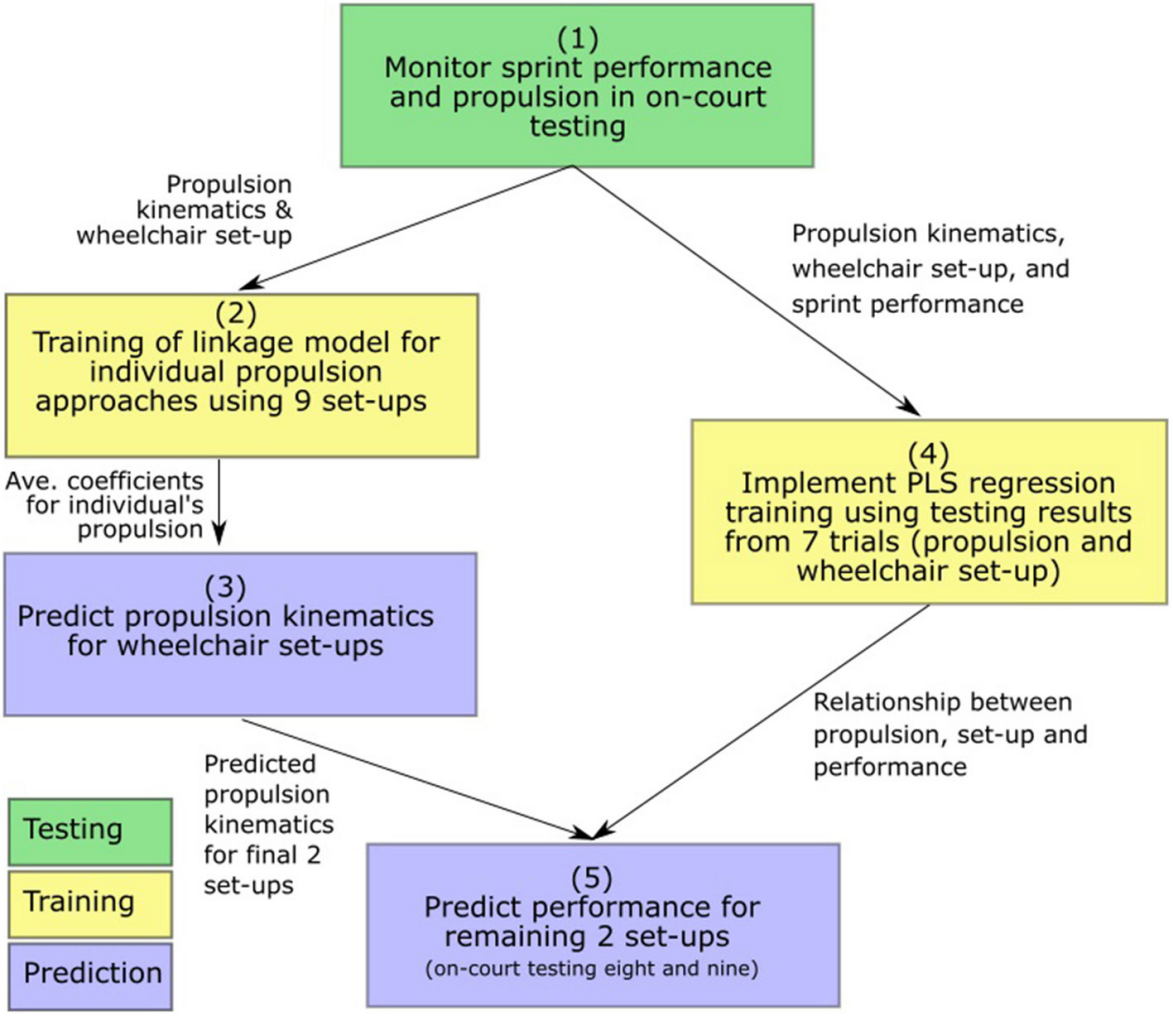
Outline of the procedure from on-court testing to performance prediction.

Performance predictions for various wheelchair set-ups from on-court testing results occurs in two main steps: (One) predicting propulsion changes when altering wheelchair set-up, and (Two) predicting performance for inputs of wheelchair set-up and propulsion kinematics. Step Two relies on propulsion prediction inputs from Step One and regression equations developed from on-court testing to predict the performance measure of sprint time. The outline of this procedure is displayed in Figure 2 and is detailed in the following sections.

#### Propulsion Prediction

A sub-maximal linkage model (Richter, 2001; Leary et al., 2012) was adapted that calculated hand contact and release angles [relative to top dead center (TDC) location, with in-front of TDC positive and behind TDC negative] based on individual anthropometrics and chair set-up. In advancing previous models to predict maximal effort propulsion (Vanlandewijck et al., 2011), the model included an additional trunk segment with trunk angular rotation (flexion) occurring about the hip position, which was assumed to be coincident with the rear of the seat and subsequently changed with seat depth and seat height. The equations for contact and release were derived to use shoulder position based on the trunk angle at hand contact and release rather than a fixed shoulder position. Trunk angular velocity (i.e., rate of progression from trunk angle at contact to trunk angle at release) was assumed to be constant throughout the stroke phase. The assumption of contact occurring when the forearm is perpendicular to the tangent of the wheel (Leary et al., 2012) was not valid for some players due to various propulsion techniques as seen in Figure 1. Players with greater trunk range of motion (in this participant group, some players with limb impairments) generally utilized an approach with a greater proportion of “push” [see Haydon et al., 2018a, where “push” is the phase of the stroke that occurs during elbow extension (Vanlandewijck et al., 2001)]. This approach requires the trunk to be in a flexed position at contact, and the forearm segment approximately parallel to the wheel tangent. For these players (detailed in Table 1 as Altered), a 90-degree addition was included for the prediction of the contact angle (Equation 1).


(1) $$\begin{array}{l} \theta_{c}=\beta \left(\tan^{-1}\left(\frac{X_{hs}-L_{ua}\sin\theta_{TI}+L_{fa}\sin\left(90{}^{\circ}-\theta_{TI}\right)}{Y_{hs}-L_{ua}\cos\theta_{TI}+L_{fa}\cos\left(90{}^{\circ}-\theta_{TI}\right)}\right)\right) \end{array}$$

Where β is a contact coefficient varied from −0.5 to 1.5 [a coefficient of 1 means the assumption of hand contact (perpendicular/parallel) is true; discussed in more detail below]; θ_*c*_ is the hand contact angle; *X*_*hs*_ is the horizontal position of the shoulder relative to the wheel axle; *Y*_*hs*_ is the vertical position of the shoulder relative to the wheel axle; *L*_*ua*_ and *L*_*fa*_ are the length of the upper arm and forearm, respectively; and θ_*TI*_ is the initial trunk angle. Anthropometric measures were completed with the support of a physiotherapist familiar with the athletes and adapted to suit the needs of each individual as per their impairment. This enabled the prediction of contact and release angles based on an individual player's anthropometrics and chair parameters (seat height, seat depth, and seat angle). As mentioned above, the seat angle setting influenced the trunk position at contact and release for each of the first three strokes and hence the trunk angles were linked with corresponding seat angle measures from testing.

The contact coefficient accounts for variation from the assumption that contact occurs when the forearm segment is perpendicular (or parallel for some players), with the coefficient being 1 when the assumption is true. This allows individual propulsion approaches to be accounted for within the overarching assumptions. During analysis of the nine set-ups tested, a contact coefficient was determined (to two decimal places) for an individual for each of the first three strokes that minimized the error between measured and predicted angles from the above equation. A contact coefficient for each of the first three strokes was then set for future predictions by averaging across the nine set-ups. A similar process was used to determine release angle coefficient for each of the three strokes using the prediction equation from previous work (Leary et al., 2012), with release angle defined as when the forearm is parallel to the wheel tangent and the trunk at most flexed position; this differs to the altered contact angle as the shoulder is now at the most forward position (due to trunk flexion). This approach not only accounts for differences across individuals, but also across the first three strokes within a sprint which have been shown to differ in accelerations from standstill (Haydon et al., 2018a). Despite the potential asymmetry present in WCR propulsion (Goosey-Tolfrey et al., 2018), this process combined left and right propulsion kinematics to reduce the impact of any outliers in coefficient calculations. The use of a single coefficient for each stroke also assumes that a player would not substantially alter their propulsion technique across wheelchair set-ups.

#### Performance Prediction

The experimental data was analyzed using a Partial Least Squares (PLS) regression. These included thirteen input variables: seat height, seat depth, seat angle, tire pressure, contact angles for the first three strokes, releases angle for the first three strokes, and the push angles for the first three strokes. The predicted variable was the sprint time. These regression approaches were trained independently in Matlab (using the *plsregress* function, Mathworks, 2017b), with the first seven of the nine set-ups from experimental testing used to train the prediction methods (within typical training-test ratios of 70–30% and 80–20%). The number of PLS components typically used in the function was set at five based on assessments of explained variance, with selection made once explained variance appeared to plateau. The number of components was adapted for each athlete depending on these results (an exemplar plot of PLS components and explained variance for Player 4 is provided in Supplementary Material, as well as the number of components and explained variances for all athletes). The performance of the prediction method was then assessed using the final two set-ups from experimental testing for each athlete. While set-up parameter values (i.e., seat height, seat depth) were matched with those from experimental testing, the prediction approach was implemented using the predicted propulsion kinematics rather than measured kinematics to ensure a true prediction from set-up to performance. The method of progression from on-court testing to performance prediction is outlined in Figure 2.

### Statistics

Mean (and standard deviations) were calculated for the difference between experimental and modeling kinematic results for each player (*n* = 8) and each stroke (*n* = 3), resulting in 24 strokes for contact and release. The ability to predict propulsion kinematics and performance was typically investigated at an individual level, with results focusing on obvious trends within these. To support this, Welch's *t*-test (for unequal variance) using an alpha of 0.05 before a Bonferroni correction (alpha adjusted to 0.008 due to six comparisons—contact angles compared with other contact angles, release angles compared with other release angles) were completed across contact and release angle differences for each stroke.

For modeling assessment, no statistical analysis was completed due to the small sample size (only two comparisons for each player) and interest in how the modeling performed at an individual level. Assessments were made from the magnitude and direction of difference between experimental and modeling performance measures.

## Results

### Propulsion Prediction

**Figure 3:**
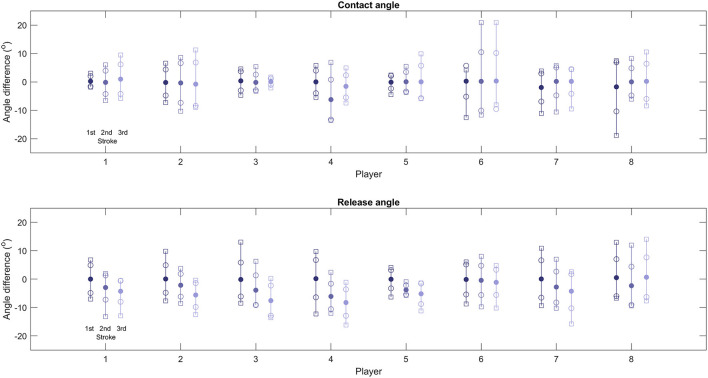
Contact and release angle prediction differences from testing results. The first three strokes for each player are presented on individual bars, with each bar containing the mean difference (filled circle), the standard deviation (open circle), and minimum and maximum differences from testing results (open squares).

For each player, kinematic data was recorded for the first three strokes with two successful trials per player (eight participants, hence 24 mean stroke results). The kinematics for each stroke were calculated and summary statistics determined for the differences between measured and predicted contact and release angles (Figure 3). Mean values suggest contact angles could be predicted with differences less than 0.5° for 18 of 24 (75%) contacts. However, the maximum differences between a measured and predicted contact angle varied by greater than 10° for 9 of 24 (37.5%) of these contacts. There were no significant differences in contact angle prediction between the three strokes. Furthermore, mean release angle prediction differences increase during later strokes after the sprint start, with significantly less error between experimental and modeling release angle prediction for stroke one compared with strokes two (release angle one: 0.05 ± 5.29°; release angle two: −3.07 ± 4.80°; *p* < 0.001) and three (release angle three: −4.55 ± 5.46°; *p* < 0.001). Maximum differences were also greater for the release angles compared with contact angles for the majority of players. There were no obvious trends for contact predictions when considering the altered contact equation (Players 1, 3, and 5 as noted in Table 1) compared with the contact prediction from previous linkage models. Specific experimental and modeling propulsion kinematic results are provided in Supplementary Material.

### Performance Prediction

**Figure 4:**
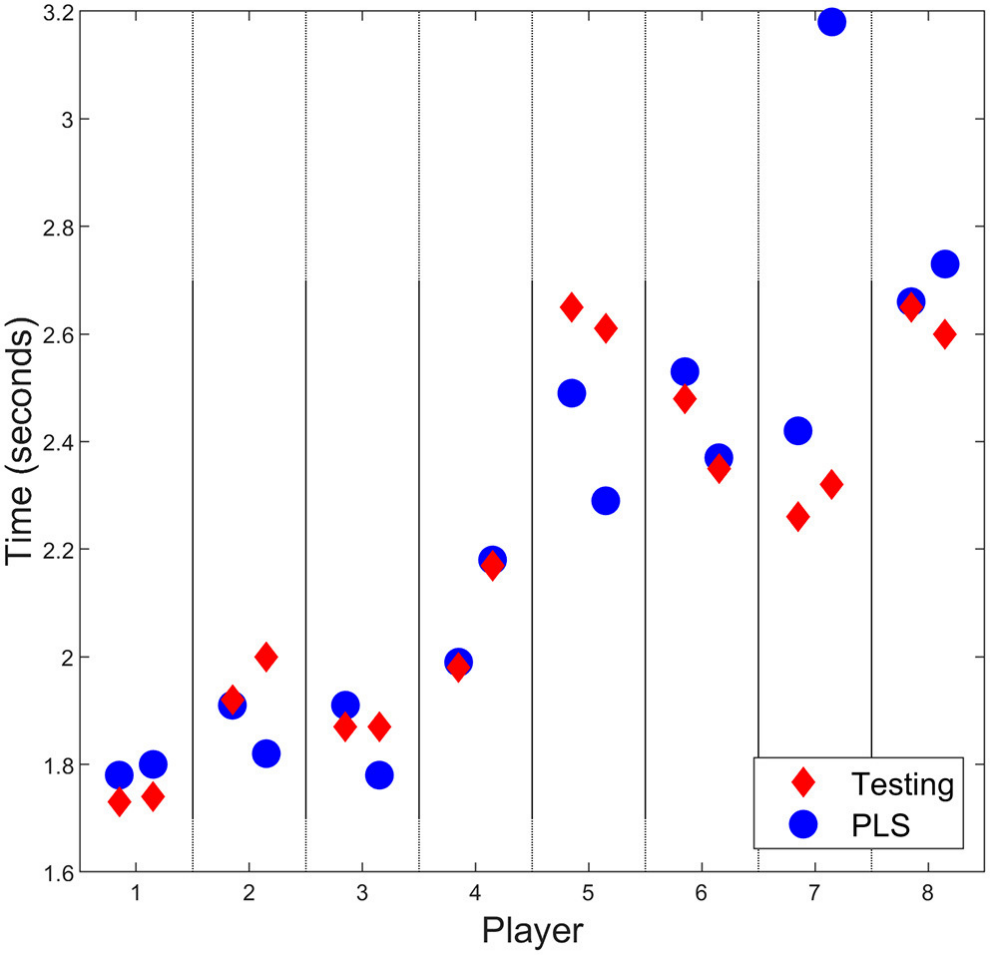
Comparison of sprint times from testing and the regression approach for all players. Most predictions follow the testing data closely, with largest differences seen in Players 5 and 7.

Sprint time predictions were calculated using chair set-up parameters and predicted propulsion angles as inputs to the PLS regression approach. Comparisons with actual (measured) sprint time for the two set-ups that were not included during training of the regression model are presented in Figure 4. Mean (±SD) sprint performance prediction error for both set-ups across all players was 0.04 (±0.25) seconds; with a minimum difference of 0.01 s (Player 4) and a maximum of 0.87 s (Player 7, set-up 2). All players, excluding Players 5 and 7, had average prediction errors of less than 0.1 s.

## Discussion

Modeling of wheelchair propulsion has the potential to minimize the amount of testing required whilst maintaining the ability to detect changes in propulsion and performance. This study investigated the ability of a linkage model to predict propulsion kinematics for a range of WCR players and use these results to predict performance using PLS regression. On-court testing captured propulsion kinematics and performance across nine set-ups using an adjustable wheelchair. Propulsion prediction equations were developed using all nine on-court testing set-ups to allow for contact and release angles for the first three strokes to be predicted for any wheelchair set-up for an individual player. A PLS regression approach was trained using seven on-court testing set-ups, leaving two for assessment of the performance prediction method. For these final two set-ups, the propulsion prediction equations were applied to provide “predicted” rather than on-court kinematics, with the PLS regression model then producing a performance time that could be compared with on-court results.

Mean values for contact angle predictions were typically similar to on-court testing results (75% within 0.5°); however, maximum differences for each player can vary substantially with the mean results impacted by the casual summation of positive and negative errors. These large differences likely occur due to the assumption that a player will attempt to employ the same propulsion technique regardless of their wheelchair set-up—evident by using an average coefficient from all nine set-ups. This assumption was a limitation of this work as players can be expected to adapt their propulsion to the specific set-up; however, it was unclear how players would adapt their technique, hence the use of the average coefficient. Changes in propulsion approach between set-ups were therefore not accounted for which likely resulted in the large differences. Mean prediction error for release angle increases after the first stroke following a sprint start for most of the players (release angle one: 0.05 ± 5.29°; release angle two: −3.07 ± 4.80°; release angle three: −4.55 ± 5.46°; *p* < 0.01 when comparing release angle one with release angle two and three). For stroke one, mean prediction error is less than 0.51° for all players and less than 0.16° for 7 of 8 players. However, for the third stroke, only 2 players had an absolute mean prediction error less than 4.27°, with a maximum error of 8.25°. This likely occurs as the magnitudes or release angles are typically larger than those of the contact angles (i.e., contact angles can vary from −45° to +15°, compared with release angles which often vary from +70° to +105°; Haydon et al., 2018a). Using an average coefficient in the calculation is therefore troublesome as slight changes to propulsion technique result in larger differences in the predicted release angle. For example, Player 8 had the smallest error for release angle estimation for the third stroke, and this player displayed the smallest release angles. This is potentially due to the variations in the coefficient value having less of an effect on the magnitude of the error—although this case does not confirm the hypothesis across the wider group. Both experimental and modeled kinematics were considered in the sagittal plane only, which is a simplification of the real-world behavior.

Regression prediction results varied between players; predicted results matched on-court testing results for some players (Vanlandewijck et al., 2011; Mason et al., 2013; Haydon et al., 2019) but were inconsistent for others (Players 2, 3, 5, 7). Player 4's results display the most potential for continued use of this approach. Despite large differences in on-court performance time in set-ups eight and nine, these changes in performance are predicted within 0.01 s by the model. This is likely influenced by a consistent relationship between wheelchair set-up, propulsion kinematics, and performance. These relationships refer to the influence changing parameters has on sprint time: in a consistent relationship, increasing contact angle is likely to have the same effect on sprint time in all set-ups. The development of this relationship occurs in the regression training (on the first seven set-ups), with the impact of wheelchair set-up and propulsion likely consistent in the tested (final two) set-ups. Player 4 regression was able to explain a high percentage of the variance, hence the ability of the model to predict performance. However, this is one case out of eight from testing; this alone does not support continued use of this approach. Although predicted performance times for Players 1 and 6 do not match as accurately, the trend is of comparable magnitude and direction. As this approach is proposed as a method to assess the effect of various wheelchair set-ups, the ability to detect changes in performance is critical. Players 2 and 3 show occasions where the regression model was poor in predicting changes in performance despite supposedly showing a high percentage of explained variance based on training for the first seven set-ups. The PLS regression approach predicted improved performance for Player 2's set-up nine, but decreased performance was evident in on-court testing. Similarly, Player 3 had similar performances in set-ups eight and nine, but regression predictions expected performance to vary by 0.13 s. Player 5 prediction did not align with performance times (average prediction error across two set-ups of 0.24 s), with performance underestimated substantially—although a slight change in prediction and on-court performance is evident. As above, this is likely due to changing relationships between wheelchair set-up and propulsion, which is emphasized for this athlete due to their lack of experience in comparison with other players. Player 7 predicted results showed minimal relationship with on-court results. Both predictions substantially overestimated the performance time, with set-up nine out by 0.87 s. For the on-court performance times for Player 7 (~2.3 s), this amount of error is clearly unacceptable. These prediction variations likely relate to regression training approaches not aligning with the relationships for tested set-ups. Greater variation in these relationships (i.e., increasing contact angle does not consistently improve/decrease sprint performance) makes performance predictions difficult; this training phase can be improved by including greater amounts of relevant data, such as individual activity limitation, however this is often difficult to achieve in practice. While propulsion prediction shows potential for some athletes, developing regression relationships that translate to on-court performance is difficult due to changing propulsion techniques. Increasing data capture would allow for stronger relationships to be determined, improving this capability, however this would require substantial time and effort. An activity that allows for simpler data capture and has clearer translation to performance measures may provide a valuable tool to further investigate the capabilities of this approach.

This wheelchair performance assessment relies on two distinct sections of prediction for changing wheelchair set-ups: (i) propulsion kinematics and (ii) sprint time performance. Propulsion kinematics were predicted based on a linkage model, with fixation about the hip an extension on previous models (Richter, 2001; Leary et al., 2012). Assessment of maximal effort propulsion from standstill in WCR requires consideration of trunk motion—due to trunk motion accompanying force generation (Vanlandewijck et al., 2011)—and player specific approaches due to the substantial variations in activity limitation across classifications (Haydon et al., 2018a). The PLS regression approach can then be trained using on-court testing to produce a prediction method based on inputs of wheelchair configuration and propulsion kinematics—allowing a greater number of potential set-ups to be investigated for players with reduced amounts of on-court testing. For this to be effective, both propulsion kinematics and performance times should be considered and be able to consistently identify small, meaningful changes. After completing on-court testing, this modeling approach can be implemented to identify set-ups of further interest. These set-ups could be replicated on-court to confirm findings, giving the player more detailed information on the effect of altering their wheelchair set-up prior to making chair modifications which can be expensive in both cost and time commitment (Haydon et al., 2019). This improves upon current implemented approaches, where small adjustments to wheelchair parameters are often made over long periods of time, which can result in players only achieving set-ups they are comfortable with (and are nearer to optimal for performance) after many years in the sport (Fletcher et al., 2021).

The linkage model used in this study was investigated as it simplifies the model of an individual (particularly with a focus on 2D kinematics, rather than more realistic 3D kinematics), resulting in a reduction in time for development and processing. The model presented in this study is an adaption to a previous model that has been successfully used to link measured kinematics with propulsion measures (Richter, 2001; Leary et al., 2012). This adaption has been added to account for atypical variations in technique exhibited by the athletes, particularly those with trunk function who are able to lean forward and “push” on the wheel/pushrim during maximal effort propulsion. While an important adaption to include for these athletes, the added features of the new model should be considered a minor addition to the original model. The results show that whilst this linkage model approach might be appropriate for some cases, it is unlikely to be suitable for all athletes; some are likely to require more detailed models that greater reflect their activity limitation (McErlain-Naylor et al., 2021). This may be the case for athletes with greater activity limitation (lower classification scores), who are less able to adapt technique to wheelchair set-up. Due to limitations in wheelchair design, lower classification scores were under-represented in this work as the majority used a smaller wheel size than was possible with the adjustable wheelchair (lower classifications often use 24-inch wheels (Goosey-Tolfrey et al., 2018) compared with 25-inch wheels for the adjustable wheelchair). Musculoskeletal models can potentially account for specific muscle functions of an individual and perform more detailed propulsion assessment through incorporation of three-dimensional motion throughout multiple strokes (Lewis et al., 2018), with the ability to develop and customize musculoskeletal models improving rapidly (Dembia et al., 2020). Individual customization of the musculoskeletal models would require further processing time and more detailed on-court testing assessment including motion capture and electromyography, which is more suited to elite level athletes initially. By constraining joint ranges of motion and adapting the level of muscle activation for an individual [both of which are possible through software such as OpenSim (SimTK, 2021)], more realistic propulsion approaches can be determined for a range of set-ups. This would likely result in greater accuracy when attempting to predict performance, particularly as the user can define specific cost functions for performance and optimize for these. However, musculoskeletal models currently find it difficult to independently scale individual body segments which would limit their applicability to amputees. The selection of modeling approach should therefore consider the ability to accurately measure and replicate individual capabilities (Lewis, 2018; McErlain-Naylor et al., 2021) as well as time restraints around any prescription approach.

An additional benefit of the modeling approaches outlined is they may allow for the reduction of experience related effects on performance. Athletes may have developed a propulsion technique that is either (i) not in fact optimal for maximal sprint performance, or (ii) is highly specific to maximizing their sprint performance in their current chair set-up (Haydon et al., 2018b). A small amount of on-court testing (i.e., a familiarization period in each testing set-up prior to data capture) is unlikely to promote adaptation to a new set-up quickly enough to get a true indication of likely performance once the athlete has adapted to the new set-up. Alternatively, changes to chair set-up may perturb the propulsion coordination and increase movement variability for a short period, again impacting the testing results (Fletcher et al., 2021). Modeling, when accounting for athlete activity limitation, could remove this concern and give a greater prediction of final performance outcomes should an alternative (i.e., predicted optimal) propulsion technique be considered. Specialists in motor control and learning (i.e., skill acquisition specialists) would then be best placed to support coaches and athletes with targeted technical (learning) interventions.

Currently, this approach requires 2- to 3-h of on-court testing with various set-ups for each individual in order to measure propulsion approaches and performance. With further progression of this method, there is the possibility to markedly reduce the amount of on-court testing required, particularly if musculoskeletal models can be developed. This progression relies on increasing the number of players and therefore data on how particular classifications and impairments respond to changes in wheelchair set-up. For players of similar impairments and anthropometry there is a greater likelihood their response to changing set-ups will be similar. As regression approaches require increases in data to build their relationships and improve reliability, international collaborations are recommended to increase the pool of elite wheelchair sport athletes.

## Conclusion

The process of wheelchair prescription is currently a time-consuming process that relies heavily on player and coach experience. This study presents a method to predict propulsion kinematics based on changing wheelchair set-ups for maximal effort sprinting. To account for maximal propulsion, an equation to predict contact angle while accounting for trunk motion was developed, improving on previous methods. Regression approaches (such as PLS) can be trained using on-court testing results, and then applied with propulsion predictions to estimate sprinting performance for WCR. Results for propulsion prediction found that the assumption of a consistent propulsion approach by using an adapted linkage model may not be appropriate, particularly for release angles. Improved understanding of wheelchair prescription impact on propulsion kinematics will support further development of accurate predictions. This scoping project suggests that while the linkage model prediction of propulsion kinematics may be suitable for some athletes, others may require more detailed models (e.g., musculoskeletal) that more accurately reflect their function. Regression approaches were inconsistent in their ability to accurately predict performance changes. Player 4's performance was predicted almost exactly despite the large variations (relative to other players) present in sprint time across set-ups eight and nine, likely due to the consistent relationship between wheelchair set-up, propulsion kinematics, and performance. However, other results were unable to achieve the same accuracy, with the expected cause being the inconsistent propulsion predictions and regression relationships. Substantial further work is required in this area to improve the process of wheelchair prescription for performance, with a greater understanding of these relationships likely to have a substantial impact on wheelchair prescription and subsequent performance.

## Data Availability Statement

The raw data supporting the conclusions of this article will be made available by the authors, without undue reservation.

## Ethics Statement

The studies involving human participants were reviewed and approved by University of Adelaide Ethics Committee. The participants provided their written informed consent to participate in this study. Written informed consent was obtained from the individual(s) for the publication of any potentially identifiable images or data included in this article.

## Author Contributions

DH, RP, PG, and WR contributed to study design and data collection. DH completed primary analysis and wrote the first draft of the manuscript, with RP, PG, WR, and CH all contributing to analysis, review and writing sections of the manuscript. All authors have reviewed the manuscript and approved its submission.

## Conflict of Interest

The authors declare that the research was conducted in the absence of any commercial or financial relationships that could be construed as a potential conflict of interest.

## Publisher's Note

All claims expressed in this article are solely those of the authors and do not necessarily represent those of their affiliated organizations, or those of the publisher, the editors and the reviewers. Any product that may be evaluated in this article, or claim that may be made by its manufacturer, is not guaranteed or endorsed by the publisher.
