# Supplementary material for: Prediction of Propulsion Kinematics and Performance in Wheelchair Rugby
Source: Front Sports Act Living. 2022 Jul 7;4:856934. doi: 10.3389/fspor.2022.856934 (PMC9301377; doi:10.3389/fspor.2022.856934)
Supplement: Supplementary file 1 [file Data_Sheet_1.docx]

Prediction of propulsion kinematics and performance in wheelchair rugby

Supplementary Material

| Set Up # | Seat Height | Seat Depth | Seat Angle | Tyre Pressure |
| --- | --- | --- | --- | --- |
| 1 | A | A | A | A |
| 2 | A | B | B | B |
| 3 | A | C | C | C |
| 4 | B | A | B | C |
| 5 | B | B | C | A |
| 6 | B | C | A | B |
| 7 | C | A | C | B |
| 8 | C | B | A | C |
| 9 | C | C | B | A |

**Table S1: An example of an L9 orthogonal array, where the four parameter settings are varied. An ‘A’ setting is an increase, ‘B’ is the current’, ‘C’ is reduced for each of the parameters. As seen, each parameter setting is tested three times, and against all other parameter settings. For example, Seat Height A is tested in the first three set-ups only. As the other parameter settings are varied in these three set-ups, it is tested against A, B, and C settings for seat depth, seat angle, and tyre pressure. This allows the distinct effect of seat height A to be determined.**

Note: The testing order of set-ups was randomised throughout testing.

**Table S2: The number of components and percent explained variance from Matlab PLS regression model for each player, with the model trained on the first seven set-ups.**

|  | Player 1 | Player 2 | Player 3 | Player 4 | Player 5 | Player 6 | Player 7 | Player 8 |
| --- | --- | --- | --- | --- | --- | --- | --- | --- |
| # of PLS components | 3 | 5 | 5 | 5 | 5 | 3 | 5 | 4 |
| % Explained Variance | 83.1 | 99.8 | 99.6 | 99.8 | 98.8 | 94.4 | 96.3 | 98.2 |


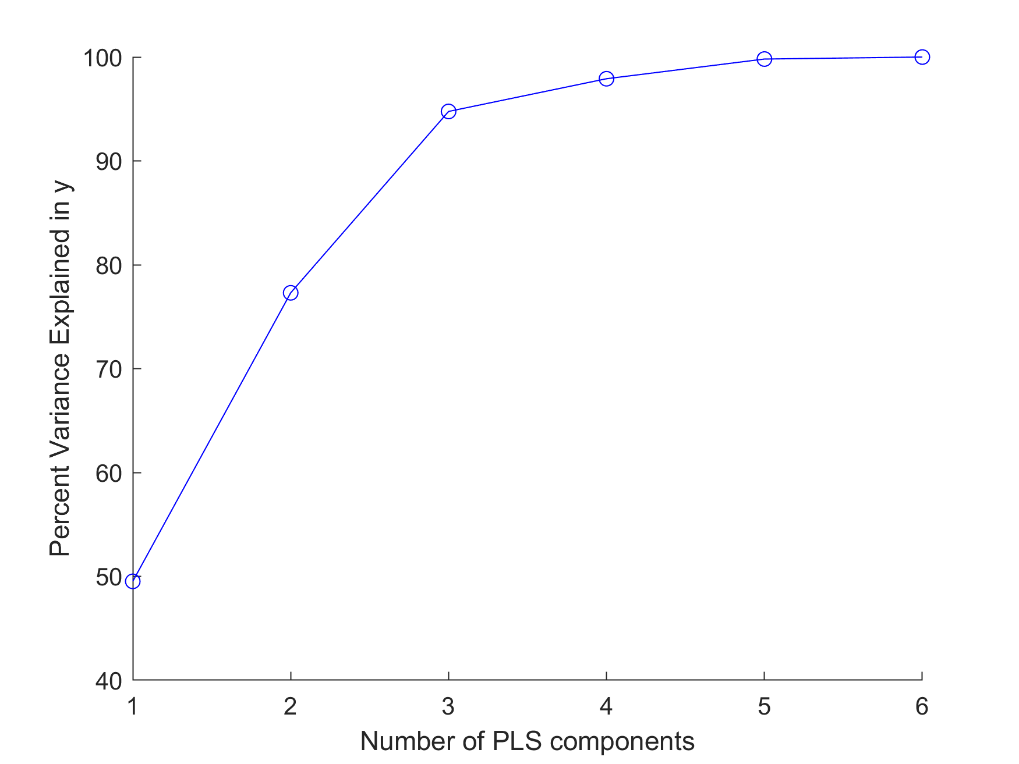


**Figure S1: An example of the number of PLS components and percent explained variance for Player 4, which displays a plateau in explained variance between 5 and 6 components.**

**Table S3: Beta Contact and release coefficients for each player.**

| Player | CA1 Coeff | CA2 Coeff | CA3 Coeff | RA1 Coeff | RA2 Coeff | RA3 Coeff |
| --- | --- | --- | --- | --- | --- | --- |
| 1 | 0.42 | 0.84 | 1.18 | 0.56 | 0.62 | 0.69 |
| 2 | 0.26 | 0.55 | 0.43 | 0.58 | 0.68 | 0.79 |
| 3 | 0.66 | 0.34 | 0.27 | 0.78 | 0.90 | 1.00 |
| 4 | 0.71 | 1.45 | 1.28 | 0.76 | 0.89 | 1.01 |
| 5 | -0.33 | 0.18 | 0.24 | 0.58 | 0.66 | 0.73 |
| 6 | 0.92 | 0.97 | 0.90 | 0.83 | 0.90 | 0.96 |
| 7 | 1.77 | 1.41 | 1.43 | 0.72 | 0.90 | 1.00 |
| 8 | 1.41 | 0.92 | 0.79 | 0.52 | 0.70 | 0.74 |

Note: Player 7 first stroke contact angle coefficient reached the initial maximum coefficient bounds (1.5), which required additional iterations to find the appropriate contact coefficient.

**Table S4: Experimental and modelling contact and release angles.**

|  | Player 1 | | | | | | | | | | | |
| --- | --- | --- | --- | --- | --- | --- | --- | --- | --- | --- | --- | --- |
|  | Experimental | | | | | | Modelling | | | | | |
| Set-up | CA1 | CA2 | CA3 | RA1 | RA2 | RA3 | CA1 | CA2 | CA3 | RA1 | RA2 | RA3 |
| 1 | 5.2 | 13.3 | 20.4 | 80.8 | 92.3 | 101.7 | 6.7 | 11.5 | 14.8 | 76.4 | 92.8 | 103.0 |
| 2 | 6.4 | 11.6 | 14.2 | 74.1 | 81.6 | 100.0 | 6.7 | 12.1 | 15.2 | 78.9 | 94.7 | 106.6 |
| 3 | 7.2 | 8.2 | 10.5 | 73.4 | 81.0 | 93.7 | 6.4 | 12.3 | 15.3 | 71.1 | 82.8 | 96.3 |
| 4 | 5.5 | 7.3 | 11.7 | 70.5 | 86.7 | 92.1 | 7.3 | 13.9 | 17.4 | 77.5 | 90.3 | 105.0 |
| 5 | 5.5 | 9.7 | 14.6 | 69.3 | 81.9 | 96.6 | 6.8 | 13.0 | 16.2 | 73.2 | 85.2 | 99.1 |
| 6 | 9.4 | 16.8 | 20.4 | 85.0 | 92.9 | 102.3 | 6.3 | 10.8 | 13.9 | 78.2 | 95.5 | 106.0 |
| 7 | 5.8 | 10.4 | 13.8 | 73.9 | 87.0 | 94.0 | 6.5 | 11.9 | 14.9 | 72.8 | 87.0 | 97.8 |
| 8 | 9.9 | 16.0 | 25.3 | 75.7 | 91.1 | 101.9 | 6.9 | 12.6 | 15.8 | 79.1 | 94.7 | 106.5 |
| 9 | 7.8 | 16.0 | 17.4 | 79.5 | 91.4 | 100.0 | 6.9 | 12.6 | 15.8 | 75.0 | 89.5 | 100.5 |
|  | Player 2 | | | | | | | | | | | |
|  | Experimental | | | | | | Modelling | | | | | |
| Set-up | CA1 | CA2 | CA3 | RA1 | RA2 | RA3 | CA1 | CA2 | CA3 | RA1 | RA2 | RA3 |
| 1 | -8.8 | -14.6 | -7.4 | 52.3 | 64.6 | 76.9 | -6.8 | -12.8 | -7.8 | 55.9 | 68.2 | 83.5 |
| 2 | -1.3 | -4.0 | 6.3 | 63.2 | 75.6 | 90.4 | -6.8 | -10.2 | -5.0 | 61.2 | 74.4 | 91.7 |
| 3 | 0.1 | -4.5 | 1.5 | 63.9 | 72.1 | 84.1 | -6.5 | -12.7 | -7.8 | 54.1 | 68.4 | 84.6 |
| 4 | -4.3 | -9.9 | -10.3 | 58.3 | 70.2 | 81.5 | -6.3 | -11.7 | -6.7 | 59.3 | 72.3 | 88.3 |
| 5 | -3.8 | -2.6 | -1.9 | 60.1 | 71.4 | 81.3 | -6.1 | -11.2 | -6.4 | 58.6 | 71.3 | 86.9 |
| 6 | -8.1 | -12.2 | -12.7 | 60.9 | 70.2 | 86.8 | -6.5 | -9.8 | -4.8 | 60.4 | 73.3 | 90.1 |
| 7 | -9.1 | -17.4 | -15.6 | 49.7 | 65.0 | 78.2 | -6.1 | -11.6 | -6.7 | 57.4 | 72.4 | 89.4 |
| 8 | -10.9 | -20.9 | -16.6 | 56.5 | 69.5 | 83.3 | -6.8 | -13.2 | -8.1 | 54.6 | 69.3 | 85.8 |
| 9 | -13.5 | -19.7 | -8.1 | 56.5 | 63.6 | 76.1 | -6.3 | -9.4 | -4.6 | 59.6 | 72.2 | 88.6 |
|  | Player 3 | | | | | | | | | | | |
|  | Experimental | | | | | | Modelling | | | | | |
| Set-up | CA1 | CA2 | CA3 | RA1 | RA2 | RA3 | CA1 | CA2 | CA3 | RA1 | RA2 | RA3 |
| 1 | -9.4 | -8.4 | -7.2 | 59.4 | 71.8 | 79.6 | -11.7 | -7.2 | -5.1 | 64.3 | 79.4 | 92.6 |
| 2 | -8.0 | -1.6 | -5.0 | 71.7 | 79.8 | 89.0 | -11.9 | -7.0 | -4.7 | 58.6 | 73.6 | 88.8 |
| 3 | -6.2 | -5.6 | -5.6 | 61.9 | 70.4 | 79.4 | -10.8 | -8.0 | -5.2 | 61.0 | 76.4 | 90.2 |
| 4 | -13.3 | -7.5 | -3.5 | 63.1 | 73.6 | 87.4 | -9.8 | -7.6 | -4.9 | 64.2 | 80.0 | 94.1 |
| 5 | -14.9 | -9.9 | -4.8 | 58.8 | 74.5 | 84.7 | -10.2 | -7.9 | -5.1 | 67.2 | 83.6 | 98.3 |
| 6 | -10.0 | -6.5 | -3.8 | 63.4 | 76.8 | 87.5 | -12.7 | -7.7 | -5.5 | 60.2 | 74.8 | 87.4 |
| 7 | -12.0 | -10.6 | -4.9 | 58.2 | 73.0 | 81.5 | -12.4 | -7.3 | -4.9 | 60.7 | 76.1 | 91.8 |
| 8 | -16.1 | -10.5 | -4.8 | 61.8 | 70.4 | 85.3 | -13.3 | -7.8 | -5.3 | 63.3 | 79.3 | 95.7 |
| 9 | -11.3 | -8.7 | -5.3 | 58.6 | 70.4 | 81.0 | -11.8 | -7.2 | -5.1 | 58.5 | 72.6 | 84.8 |
|  | Player 4 | | | | | | | | | | | |
|  | Experimental | | | | | | Modelling | | | | | |
| Set-up | CA1 | CA2 | CA3 | RA1 | RA2 | RA3 | CA1 | CA2 | CA3 | RA1 | RA2 | RA3 |
| 1 | -20.2 | -36.1 | -28.8 | 68.7 | 74.7 | 84.6 | -22.7 | -34.3 | -30.4 | 59.0 | 82.0 | 90.1 |
| 2 | -22.8 | -36.6 | -29.8 | 68.1 | 83.5 | 91.5 | -22.0 | -32.6 | -28.3 | 64.8 | 89.1 | 98.1 |
| 3 | -22.3 | -28.7 | -24.9 | 62.7 | 81.1 | 90.8 | -24.1 | -35.6 | -29.8 | 55.3 | 78.7 | 92.0 |
| 4 | -25.3 | -45.8 | -32.1 | 59.1 | 63.9 | 80.3 | -21.2 | -37.0 | -32.7 | 61.8 | 76.0 | 92.2 |
| 5 | -29.6 | -44.1 | -36.5 | 62.2 | 75.8 | 87.4 | -24.1 | -36.6 | -32.6 | 60.0 | 83.7 | 91.9 |
| 6 | -24.2 | -48.2 | -29.9 | 60.3 | 70.1 | 83.4 | -19.9 | -34.8 | -30.6 | 60.9 | 74.7 | 90.5 |
| 7 | -17.7 | -34.4 | -30.5 | 59.9 | 84.1 | 91.7 | -23.5 | -34.0 | -27.5 | 61.0 | 85.8 | 100.1 |
| 8 | -18.0 | -45.3 | -31.5 | 55.5 | 77.2 | 84.9 | -22.2 | -31.8 | -25.6 | 60.1 | 84.0 | 97.9 |
| 9 | -18.2 | -46.1 | -35.9 | 47.6 | 62.1 | 72.5 | -18.6 | -32.5 | -28.5 | 59.9 | 73.4 | 88.6 |
|  | Player 5 | | | | | | | | | | | |
|  | Experimental | | | | | | Modelling | | | | | |
| Set-up | CA1 | CA2 | CA3 | RA1 | RA2 | RA3 | CA1 | CA2 | CA3 | RA1 | RA2 | RA3 |
| 1 | 9.9 | -8.4 | -11.6 | 49.8 | 53.3 | 59.3 | 10.6 | -5.8 | -6.3 | 45.7 | 59.1 | 65.7 |
| 2 | 12.7 | 0.0 | 3.6 | 49.4 | 53.3 | 57.1 | 10.3 | -5.5 | -6.3 | 45.6 | 58.1 | 67.7 |
| 3 | 10.0 | -2.9 | 0.1 | 42.9 | 50.7 | 54.9 | 11.4 | -6.1 | -6.7 | 44.9 | 56.5 | 66.1 |
| 4 | 6.3 | -9.4 | -12.1 | 37.9 | 51.2 | 59.8 | 10.7 | -5.8 | -6.2 | 44.2 | 55.6 | 65.0 |
| 5 | 10.3 | -8.4 | -6.3 | 42.7 | 53.0 | 62.7 | 9.6 | -5.1 | -5.8 | 44.9 | 57.1 | 66.5 |
| 6 | 8.5 | -8.2 | -11.9 | 49.2 | 59.7 | 67.0 | 10.6 | -5.8 | -6.1 | 48.5 | 62.3 | 69.3 |
| 7 | 11.3 | -4.0 | -5.6 | 49.1 | 58.1 | 67.3 | 9.6 | -5.1 | -5.7 | 47.7 | 60.3 | 70.1 |
| 8 | 11.4 | -6.6 | -8.5 | 44.5 | 53.7 | 62.5 | 10.1 | -5.4 | -5.8 | 43.5 | 54.6 | 63.9 |
| 9 | 11.6 | -1.5 | -2.0 | 47.0 | 57.1 | 65.2 | 9.9 | -5.4 | -5.7 | 47.8 | 61.2 | 68.0 |
|  | Player 6 | | | | | | | | | | | |
|  | Experimental | | | | | | Modelling | | | | | |
| Set-up | CA1 | CA2 | CA3 | RA1 | RA2 | RA3 | CA1 | CA2 | CA3 | RA1 | RA2 | RA3 |
| 1 | -43.9 | -23.0 | -19.9 | 52.0 | 60.0 | 59.9 | -45.7 | -43.9 | -40.9 | 53.5 | 57.2 | 62.3 |
| 2 | -37.4 | -34.7 | -30.8 | 47.6 | 57.2 | 65.7 | -41.1 | -43.2 | -40.5 | 56.4 | 61.1 | 67.1 |
| 3 | -40.4 | -37.3 | -35.1 | 51.3 | 61.1 | 61.6 | -44.4 | -45.3 | -41.2 | 47.2 | 53.1 | 57.6 |
| 4 | -43.5 | -44.0 | -45.9 | 59.2 | 54.0 | 61.2 | -43.7 | -41.8 | -38.9 | 53.1 | 56.7 | 61.7 |
| 5 | -41.4 | -52.5 | -43.6 | 56.8 | 61.2 | 63.1 | -43.3 | -43.9 | -39.8 | 52.9 | 59.2 | 64.0 |
| 6 | -49.4 | -49.8 | -48.9 | 55.4 | 55.8 | 60.3 | -45.3 | -46.1 | -41.8 | 53.2 | 59.6 | 64.5 |
| 7 | -39.7 | -49.3 | -47.3 | 42.2 | 45.2 | 50.3 | -42.7 | -44.8 | -42.0 | 50.7 | 55.0 | 60.5 |
| 8 | -43.5 | -51.1 | -45.4 | 54.6 | 58.9 | 67.4 | -47.8 | -46.1 | -42.9 | 53.8 | 57.5 | 62.7 |
| 9 | -53.2 | -54.4 | -48.1 | 50.8 | 56.8 | 60.6 | -40.7 | -42.7 | -40.0 | 50.4 | 54.6 | 60.1 |
|  | Player 7 | | | | | | | | | | | |
|  | Experimental | | | | | | Modelling | | | | | |
| Set-up | CA1 | CA2 | CA3 | RA1 | RA2 | RA3 | CA1 | CA2 | CA3 | RA1 | RA2 | RA3 |
| 1 | -44.2 | -37.3 | -38.6 | 54.4 | 61.9 | 61.4 | -45.2 | -40.2 | -37.9 | 52.2 | 71.0 | 77.2 |
| 2 | -49.2 | -39.1 | -37.5 | 52.7 | 66.3 | 77.9 | -52.5 | -44.8 | -41.6 | 51.3 | 66.5 | 76.3 |
| 3 | -46.7 | -36.1 | -36.3 | 51.9 | 70.5 | 77.8 | -50.6 | -41.0 | -39.2 | 51.1 | 68.9 | 78.9 |
| 4 | -53.3 | -40.2 | -39.3 | 52.0 | 66.0 | 74.8 | -48.4 | -39.2 | -37.4 | 50.6 | 68.2 | 78.1 |
| 5 | -57.2 | -48.0 | -45.2 | 40.8 | 62.2 | 67.5 | -46.1 | -37.4 | -35.7 | 50.1 | 67.4 | 77.1 |
| 6 | -46.1 | -44.5 | -38.6 | 40.1 | 56.3 | 66.8 | -46.8 | -41.1 | -39.1 | 48.6 | 66.5 | 72.3 |
| 7 | -53.0 | -42.2 | -40.0 | 50.0 | 64.4 | 72.7 | -49.0 | -42.2 | -38.7 | 54.4 | 70.3 | 80.4 |
| 8 | -49.3 | -37.2 | -31.7 | 62.6 | 77.3 | 76.8 | -43.1 | -38.4 | -36.1 | 51.7 | 70.3 | 76.4 |
| 9 | -50.5 | -41.0 | -36.7 | 56.9 | 64.8 | 78.0 | -50.2 | -42.9 | -39.8 | 50.8 | 65.8 | 75.4 |
|  | Player 8 | | | | | | | | | | | |
|  | Experimental | | | | | | Modelling | | | | | |
| Set-up | CA1 | CA2 | CA3 | RA1 | RA2 | RA3 | CA1 | CA2 | CA3 | RA1 | RA2 | RA3 |
| 1 | -62.6 | -42.6 | -37.2 | 44.6 | 66.3 | 77.7 | -69.0 | -50.9 | -43.2 | 44.7 | 64.4 | 72.5 |
| 2 | -65.6 | -50.0 | -40.6 | 38.5 | 57.3 | 68.5 | -70.6 | -52.1 | -43.6 | 45.3 | 66.8 | 74.8 |
| 3 | -61.0 | -50.3 | -47.1 | 42.3 | 67.6 | 71.9 | -68.5 | -52.3 | -43.6 | 48.4 | 72.5 | 79.6 |
| 4 | -68.2 | -45.1 | -30.8 | 57.7 | 76.1 | 85.9 | -65.9 | -48.7 | -41.3 | 44.7 | 64.1 | 71.8 |
| 5 | -71.0 | -56.1 | -50.1 | 45.7 | 67.2 | 77.0 | -65.4 | -50.1 | -41.7 | 48.2 | 71.6 | 78.4 |
| 6 | -68.0 | -51.1 | -43.1 | 53.6 | 65.0 | 79.8 | -62.4 | -47.9 | -39.8 | 47.9 | 70.6 | 77.2 |
| 7 | -85.6 | -53.9 | -45.3 | 48.6 | 66.3 | 78.5 | -66.7 | -49.5 | -41.2 | 50.0 | 73.2 | 81.7 |
| 8 | -67.7 | -52.0 | -40.1 | 47.3 | 65.0 | 76.0 | -67.5 | -49.9 | -41.7 | 45.1 | 66.2 | 73.9 |
| 9* |  |  |  |  |  |  | -69.5 | -51.1 | -43.5 | 40.2 | 58.1 | 65.4 |

Note: Player 8, Set-up 9 propulsion kinematics were not captured due to a technology failure discovered during processing.
